# First Whole-Genome Shotgun Sequence of a Promising Cellulase Secretor, *Trichoderma koningiopsis* Strain POS7

**DOI:** 10.1128/genomeA.00823-17

**Published:** 2017-09-14

**Authors:** María Lorena Castrillo, Gustavo Ángel Bich, Carlos Modenutti, Adrián Turjanski, Pedro Darío Zapata, Laura Lidia Villalba

**Affiliations:** a Laboratorio de Biotecnología Molecular, Instituto de Biotecnología Misiones (InBioMis), Facultad de Ciencias Exactas Químicas y Naturales, Universidad Nacional de Misiones, Posadas, Misiones, Argentina; b Instituto de Química Biológica, Facultad de Ciencias Exactas y Naturales (IQUIBICEN), Universidad de Buenos Aires, Buenos Aires, Argentina; c Consejo Nacional de Investigaciones Científicas y Técnicas (CONICET), Buenos Aires, Argentina

## Abstract

*Trichoderma koningiopsis* strain POS7 produces significantly large amounts of cellulase enzymes in solid-state fermentation. The Illumina-based sequence analysis reveals an approximate genome size of 36.6 Mbp, with a G+C content of 48.82% for *T. koningiopsis* POS7. Based on *ab initio* prediction, 12,661 coding genes were annotated.

## GENOME ANNOUNCEMENT

Cellulolytic fungi, such as many *Trichoderma* species, play an important role in natural ecosystems, participating in the transformation of cellulose (1). Therefore, many efforts have been made toward the isolation and mass multiplication of microorganisms of this genus to produce cellulase enzymes with higher specific activity and outstanding efficiency (2, 3). The *Trichoderma koningiopsis* POS7 strain of this study was found to produce significantly large amounts of cellulase enzymes in solid-state fermentation, with high enzymatic stability (4). Looking at these prospects, we aimed to sequence the genome of a promising cellulase secretor strain and to gain detailed insights into its genomic features. We report for the first time, to our knowledge, the genome sequence of the *T. koningiopsis* species.

*T. koningiopsis* strain POS7 was collected from a forest environment of Misiones (Argentina) (27°24′31.8″S, 55°53′48.5″W). Genomic DNA from *T. koningiopsis* POS7 was extracted according to the protocol reported previously (5). Genomic DNA library construction and draft genome sequencing were performed by Macrogen Co. (South Korea) using the Illumina MiSeq system. A genomic DNA library was prepared using a rapid shotgun library. Quality control procedures removed DNA spike-in, artifacts, and ambiguous or low-quality reads. Paired ends having at least 90% of bases with a quality score greater than or equal to Q20 were filtered before assembly. These sequences were assembled *de novo* using the IDBA software package (6), and SSPACE was used to assemble the sequences in scaffolds (7). To predict genes in the *T. koningiopsis* POS7 genome, we used an *ab initio* gene predictor, geneID (8), which is specifically trained for this genome, and one homology-based gene predictor, Exonerate (9). Using a heuristic approach implemented in a homemade pipeline, we combined all predicted gene models to produce a nonredundant set of genes, in which a single best-gene model per locus was selected on the basis of sequence similarity to known proteins. We annotated and classified genes according to Gene Ontology (GO), EC numbers, and eukaryotic orthologous groups (KOGs).

The resulting genome sequence of *T. koningiopsis* POS7 has an estimated size of 36.6 Mb and a GC content of 48.82%. To accomplish the next-generation sequencing libraries, a rapid shotgun library was sequenced, resulting in 7,773,936 paired reads (36,586,254 bp), with an approximate insert size of 100 bp.

The reads were assembled in 147 scaffolds, with a scaffold *N*_50_ value of 1.75 Mb. This representative set included 12,661 protein-coding genes. The majority (68%) of the predicted genes contained multiple exons, with an average of 2.59 exons per gene. The average gene density, similar to that of most of the larger scaffolds, was 2.9 kb per gene. The average protein length was 450 amino acids. In total, 12,642 (99%) gene models were predicted to be complete. Approximately 83.5% of the predicted proteins showed sequence similarity to other proteins, primarily from fungi. We assigned GO terms to 7,509 (59.3%) of the predicted *Trichoderma* proteins. We also assigned 3,554 (28%) proteins to KOG clusters and 1,146 distinct EC numbers to 3,204 (25%) proteins.

### Accession number(s).

This whole-genome shotgun project has been deposited at GenBank under the accession number MRBD00000000. The version described in this paper is the first version, MRBD01000000.
